# On-line Italian register for severe/non-controlled asthma

**DOI:** 10.1186/2045-7022-3-S1-P16

**Published:** 2013-05-03

**Authors:** Megon Bresciani, Vincenzo Bellia, Floriano Bonifazi, Roberto De Marco, Francesco Forastiere, Pierluigi Paggiaro, Gianni Passalaqua, Fabrizio Spinozzi, Giovanni Viegi

**Affiliations:** 1 Consiglio Nazionale delle Ricerche, Istituto di Fisiologia Clinica, Italy; 2 Università degli Studi di Palermo, Malattie dell’Apparato Respiratorio, Italy; 3 Azienda Ospedaliera Universitaria Ospedali Riuniti di Ancona, Dipart Malattie Respiratorie e Immunoallergiche, Italy; 4 Università di Verona, Unità di Epidemiologica e Statistica Medica, Italy; 5 Regione Lazio, Dipartimento di Epidemiologia, Italy; 6 Università di Pisa, Dipartimento Cardio Toracico e Vascolare, Italy; 7 Università di Genova, Dip Medicina, Malattie Allergiche e Respiratorie, Italy; 8 Università di Perugia, Dipartimento di Medicina Clinica e Sperimentale, Italy

## Background

Severe/Non-controlled asthma (SNCA) is a crucial challenge for physicians and a socio-economic burden for National Health Services (NHS). In Italy more than 50% of costs for asthma (1-2% of total NHS expenditure) are due to SNCA and moreover, within the European Community Respiratory Health Survey, Italy was the country with the lowest % of ICS daily use (29%) and with the highest % of subjects with uncontrolled asthma despite treatment (67% vs an overall European mean of 47%). Despite few data from very selected centers, in our country a precise estimate of the epidemiological figures and the disease related costs for SNCA is not available. Thus, we aimed at instituting of an on-line Italian register for SNCA (Registro Italiano Asma Grave e Non Controllata) published on the website of the Italian Health Agency (Istituto Superiore di Sanità -ISS) and financed by the Italian Drug Agency (Agenzia Italiana per il Farmaco **–** AIFA). Objectives To assess in general population and in clinical settings the effectiveness of therapeutic strategies for SNCA, defined in accordance with GINA guidelines. Secondary objectives are: obtain clinical indicators of diagnostic and therapeutic appropriateness for SNCA, evaluate direct and indirect costs of this disease, obtain continuous monitoring of SNCA patients, some epidemiological figures and finally disseminate the use of the register among Italian clinical centers.

## Methods

The register is composed of 9 different sections and a follow-up section accessible, at present, only to the 8 centers participating in the AGAVE (severe asthma: epidemiological and clinical cohorts follow up; therapeutic appropriateness and outcome assessment) project, financed by AIFA. These centers will use the register to enter clinical data from their SNCA patients. All patients with a diagnosis of severe asthma since at least one year and all uncontrolled asthma despite regular treatment according to GINA step 3 or 4 are eligible. Patients will be selected from prospective and retrospective longitudinal studies of pre-existing cohorts of different age groups and different geographical areas. Follow-up will be performed every 6 months for 24 months. The estimated number of recruited patients from the clinical setting is 375, while 500 patients are expected from the epidemiological setting.

## Conclusions

Our goal is to collect more data on SNCA patients and disseminate the on-line register for use by National Health Service physicians.

